# Uncertainty-aware machine learning for core-loss background subtraction in EELS

**DOI:** 10.1038/s41524-026-02145-3

**Published:** 2026-05-26

**Authors:** Bart van der Wielen, Jeroen J. M. Sangers, Samuel Mañas-Valero, Juan Rojo, Sonia Conesa-Boj

**Affiliations:** 1 Kavli Institute of Nanoscience, Delft University of Technology, Delft, The Netherlands; 2 Nikhef Theory Group, Amsterdam, The Netherlands; 3 Department of Physics and Astronomy, Vrije Universiteit Amsterdam, Amsterdam, The Netherlands

**Keywords:** Materials science, Physics

## Abstract

Quantitative electron energy-loss spectroscopy (EELS) critically depends on accurate background subtraction, yet standard approaches rely on deterministic parametric fits and provide no statistically rigorous uncertainty estimates. This limitation is particularly severe in the core-loss regime, where the background must be extrapolated into the ionisation-edge window, hindering reliable and automated analyses. Here, we introduce a physics-informed, uncertainty-aware machine learning framework that reformulates core-loss background subtraction as a statistically controlled inference problem with explicit uncertainty propagation. The method combines Monte Carlo replica generation based on empirically determined covariance matrices with neural network ensembles trained on the pre-edge region, and enforces physically consistent extrapolation via an effective-exponent constraint at the edge onset. This approach yields pixel- and energy-resolved background probability distributions, enabling direct propagation of uncertainty to derived spectroscopic observables. Closure tests on synthetic spectral images demonstrate faithful background reconstruction and well-calibrated uncertainty estimates, including in extrapolation regions. Applied to core-loss EELS spectral images of twisted CrSBr, the framework reveals nanoscale, stacking-correlated modulations of Cr L_2,3_ white-line intensities that significantly exceed propagated uncertainties. More broadly, this work provides an automation-ready pathway for quantitative, uncertainty-aware core-loss EELS analysis across diverse materials systems.

## Introduction

Electron energy-loss spectroscopy (EELS) in the scanning transmission electron microscope (STEM) provides direct access to electronic structure, bonding, and elemental composition with nanometre and even atomic spatial resolution ^1–5^. In the core-loss regime, ionisation edges encode element-specific information such as oxidation states, coordination environments, and orbital occupancy, enabling quantitative mapping of electronic structure in complex and low-dimensional materials ^6–8^. However, the extraction of such information critically depends on accurate background subtraction, as the relevant spectral features are superimposed on a steeply decaying and spatially varying background.

Conventional core-loss background (CLB) subtraction approaches typically rely on deterministic parametric models, most commonly power-law or exponential fits applied to a user-defined pre-edge window ^9,10^. While these methods are widely used and computationally efficient, they are sensitive to fitting-range selection, can be biased by residual signal or plural scattering, and fundamentally provide a single background estimate without statistically rigorous uncertainty quantification. More advanced approaches, including model-based quantification, likelihood-based ^11^, multi-region fitting ^12^, and principal component analysis (PCA) based noise filtering ^13^, benefit from improved robustness and reproducibility but continue to treat the background as a static object. As a result, uncertainties are rarely propagated to derived quantities such as integrated edge intensities or white-line ratios, limiting the reliability of quantitative interpretation, particularly when subtle spectral contrast is sought.

These limitations become especially severe in the core-loss regime, because background subtraction is intrinsically an extrapolation problem: the background must be inferred inside the ionisation-edge window, where no training data are available, and the spectrum contains unknown inelastic signal contributions ^4,5^. While recent machine learning (ML) approaches have been successfully applied to EELS for denoising, spectral classification, hyperspectral unmixing, and automated edge recognition ^14–17^, they generally either rely on conventional background subtraction as a preprocessing step or implicitly absorb the background into a learned representation. Consequently, they do not address the central difficulty of core-loss EELS, namely, how to perform background subtraction in a statistically controlled manner while quantifying and propagating uncertainty through extrapolation.

In this work, we address this challenge by reformulating CLB subtraction as an uncertainty-aware statistical inference problem. Building on Monte Carlo ensemble learning concepts previously applied to the low-loss EELS regime^18–21^, we extend this framework to core-loss spectroscopy, where extrapolation is unavoidable. Our approach combines Monte Carlo replica generation based on empirically determined, cluster-wise covariance matrices with neural-network ensembles trained on the pre-edge region, ensuring faithful representation of experimental noise and correlations. Crucially, we introduce a physics-informed effective-exponent constraint ^22^ that enforces physically consistent continuation of the learned background into the ionisation-edge window ^4^. This hybrid strategy, flexible where data exist and physically constrained where they do not, yields pixel- and energy-resolved probability distributions for the inferred background, enabling direct propagation of uncertainty to downstream spectroscopic observables.

## Results

### CLB subtraction framework

We formulate CLB subtraction in EELS spectrum images (EELS-SIs) as an uncertainty-aware inference problem, in which the CLB is learned from the pre-edge region and extrapolated into the ionisation-edge window with quantified confidence. The central challenge addressed here is that the signal region contains unknown inelastic contributions and therefore cannot be used for training, making extrapolation unavoidable and uncertainty estimation essential. Our approach is designed to provide not only a best-estimate background, but a statistically calibrated description of uncertainty that can be propagated to downstream spectroscopic observables.

**Fig. 1:**
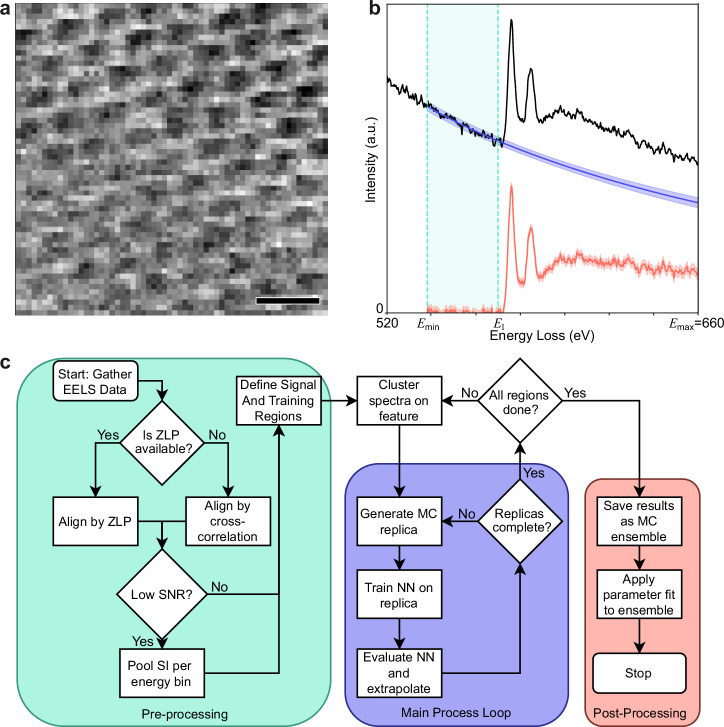
CLB subtraction procedure. **a** Representative HAADF-STEM image acquired on CrSBr layers. This HAADF-STEM image is recorded simultaneously with an EELS-SI, and each pixel corresponds to an individual EELS spectrum. The scale bar corresponds to 0.5 nm. **b** One of the spectra obtained from the EELS-SI associated with the specimen displayed in (**a**), focusing on the core-loss region between 520 and 660 eV. The black curve corresponds to the original data, the blue band to the ML model for the CLB and its associated uncertainties, and the red curve to the subtracted core-loss spectrum together with the propagated uncertainties. The ML model is trained on the background region between *E*_min_ and *E*_*I*_ and extrapolated to the signal region between *E*_*I*_ and ${E}_{\max }$. The two narrow peaks displayed correspond to the Cr *L*_2_ and *L*_3_ edges. **c** Schematic workflow summarising the CLB subtraction procedure for EELS-SI. The workflow is divided into preprocessing, ML training (main process loop), and post-processing of results.

The full workflow is summarised schematically in Fig. 1c, and representative input data are shown in Fig. 1a, b, including the simultaneously acquired HAADF-STEM image and a corresponding core-loss EELS spectrum. The procedure consists of three main stages: (i) statistical representation of experimental variability through Monte Carlo replica generation, (ii) flexible background learning in the pre-edge region using neural network ensembles, and (iii) physics-informed extrapolation into the ionisation-edge window. Together, these steps yield pixel- and energy-resolved background probability distributions rather than a single deterministic background estimate.

Prior to model training, the EELS-SIs undergo preprocessing steps including spectral alignment, optional spatial pooling to improve signal-to-noise ratio (SNR), and selection of training and signal energy windows. These steps are described in the Methods section; the definition of the training, effective-exponent, and extrapolation regions is schematically illustrated in Fig. S1, and their role in the overall workflow is indicated in the preprocessing block of Fig. 1c. In the following sections, we describe each stage of the framework in detail, before validating its statistical calibration on synthetic data and applying it to experimental core-loss EELS measurements on twisted CrSBr.

#### Clustering and Monte Carlo replica generation

A core-loss EELS-SI is represented as a three-dimensional dataset *I*^(*i*, *j*)^(*E*) defined on a spatial grid (*i*, *j*) and an energy-loss *E* (Fig. 1a). To capture systematic spectral variations across the spectrum image while keeping uncertainty propagation computationally tractable, we first compress the dataset by clustering spectra with similar integrated core-loss intensity.

For each pixel (*i*, *j*), we compute the integrated intensity in the core-loss window as

1 $${N}_{{\rm{tot}}}^{(i,j)}\equiv \mathop{\sum }\limits_{E={E}_{\min }}^{{E}_{\max }}{I}^{(i,j)}(E),\,i=1,\ldots ,{n}_{{\rm{x}}},\,j=1,\ldots ,{n}_{{\rm{y}}},$$

which provides a scalar descriptor of thickness- and density-related variations across the spectrum image. Clustering is then performed using the *K*-means algorithm applied to the logarithm of ${N}_{{\rm{tot}}}^{(i,j)}$, accommodating the wide dynamic range typical of core-loss EELS data. The clustering is defined by minimisation of the cost function

2 $${C}_{K}=\mathop{\sum }\limits_{k=1}^{K}\mathop{\sum }\limits_{(i,j)\in {S}_{k}}ln\left(\frac{{N}_{{\rm{tot}}}^{(i,j)}}{{\overline{N}}_{k}}\right),$$

where ${\overline{N}}_{k}$ denotes the centre of cluster *k*.

Within each of the *K* clusters, spectra are treated as statistically equivalent. We estimate the cluster-wise mean spectrum ${\bar{I}}_{k}(E)$ and the inter-energy covariance matrix ${\Sigma }_{k}(E,{E}^{{\prime} })$, which together define a multivariate Gaussian model for spectral fluctuations.

From this model, we generate *N*_rep_ Monte Carlo replicas per cluster according to

3 $${I}_{k,\ell }(E) \sim {\mathcal{N}}\,\left({\bar{I}}_{k}(E),\mathop{\sum }\limits_{k}(E,{E}^{{\prime} })\right),\,\ell =1,\ldots ,{N}_{\mathrm{rep}},$$

with ${\mathcal{N}}$ indicating the normal distribution, ensuring faithful reproduction of both spectral variance and inter-energy correlations.

By construction, ensemble averages and inter-energy covariances of the Monte Carlo replicas reproduce the statistical properties of the corresponding experimental clusters. This compression reduces the learning problem from $({n}_{x}\times {n}_{y}) \sim {\mathcal{O}}(1{0}^{5})$ individual spectra to $({n}_{K}\times {N}_{{\rm{rep}}}) \sim {\mathcal{O}}(1{0}^{3})$ statistically representative replicas, keeping neural-network training computationally tractable. Unless stated otherwise, we use *N*_rep_ = 1000 replicas per cluster, which is found to be sufficient to yield stable uncertainty estimates for all physical observables reported below.

#### Neural network training

For each spectrum, the measured intensity is decomposed into a CLB contribution and an inelastic signal contribution according to

4 $${I}_{\mathrm{dat}}^{(i,j)}(E)=\left\{\begin{array}{ll}{I}_{\mathrm{CLB}}^{(i,j)}(E), & E < {E}_{{\rm{I}}},\\ {I}_{\mathrm{CLB}}^{(i,j)}(E)+{I}_{\mathrm{inel}}^{(i,j)}(E), & E\ge {E}_{{\rm{I}}},\end{array}\right.\,i=1,\ldots ,{n}_{x},\,\,j=1,\ldots ,{n}_{y},$$

with an analogous separation defined for each Monte Carlo replica *I*^(*k*, *ℓ*)^(*E*).

Neural networks are trained exclusively on the pre-edge region *E* < *E*_I_, where the background is directly observed and uncontaminated by inelastic signal. Each network takes as input the energy loss *E* together with the logarithm of the integrated core-loss intensity, $ln{N}_{{\rm{tot}}}$, which labels the cluster and serves as a proxy for local thickness. Training is performed independently for each Monte Carlo replica, yielding an ensemble of neural-network models.

The training minimises a loss function ${C}_{{\rm{CLB}}}^{(\ell )}$ composed of a variance-weighted Gaussian log-likelihood term (in log-intensity) and a monotonicity penalty that suppresses unphysical positive slopes of the background:

5 $${C}_{\mathrm{CLB}}^{(\ell )}=\mathop{\sum }\limits_{k=1}^{{n}_{K}}\mathop{\sum }\limits_{E < {E}_{{\rm{I}}}}\left[\frac{{\left({\mathrm{ln}}\,{I}^{(k,\ell )}(E)-{\xi }^{(\ell )}\left(E,{\mathrm{ln}}{N}_{\mathrm{tot}}^{(k)}\right)\right)}^{2}}{{\left({\sigma }_{I}^{(k)}(E)\right)}^{2}}+\lambda \,\mathrm{ReLU}\,\left(\frac{\partial \,{\xi }^{(\ell )}\left(E,{\mathrm{ln}}{N}_{\mathrm{tot}}^{(k)}\right)}{\partial E}\right)\right],$$

where *ℓ* = 1, …, *N*_rep_ labels the Monte Carlo replicas, *ξ*^(*ℓ*)^ denotes the neural-network prediction, and ${\sigma }_{I}^{(k)}(E)$ stands for the standard deviation for the logarithm of the intensity within the replicas of the *k*-th cluster.

The dependence of *ξ*^(*ℓ*)^ on the total integrated intensity $ln{N}_{{\rm{tot}}}^{(k)}$, Eq. (1), allows the model to capture systematic thickness-related variations across clusters. The second term in Eq. (5) penalises neural-network configurations that exhibit an unphysical local increase with energy loss, thereby enforcing a monotonically decaying background during training.

#### Extrapolation to the signal region

Since the ionisation-edge window *E* ≥ *E*_I_ contains unknown inelastic signal contributions, it is excluded from neural-network training, and the learned background must be extrapolated beyond the pre-edge region. A statistically controlled extrapolation is therefore required to obtain physically meaningful background estimates in the signal region.

To enforce physically consistent behaviour during extrapolation, we match the neural-network prediction at the edge onset to a power-law decay characterised by an effective exponent. This exponent is estimated from the local logarithmic slope of the neural-network prediction in a narrow energy window immediately below the edge onset,

6 $${r}_{\mathrm{eff}}(E)\equiv -\,\frac{\partial {\mathrm{lnI}}_{\mathrm{CLB}}(E)}{\partial \mathrm{ln}E}\,\simeq \,-\,\frac{\Delta {E}_{\mathrm{bin}}}{\Delta {E}_{r}}\mathop{\sum }\limits_{{E}_{{\rm{I}}}-\Delta {E}_{r} < E < {E}_{{\rm{I}}}}\frac{\partial \xi \,\left(E,\mathrm{ln}{N}_{\mathrm{tot}}\right)}{\partial \mathrm{ln}E},$$

where Δ*E*_*r*_ denotes the width of the averaging window and Δ*E*_bin_ the energy-bin size. The choice of the effective-exponent window is discussed in the Methods section. This procedure stabilises the extrapolation while retaining sensitivity to local spectral variations learned in the pre-edge region.

The final background model for each Monte Carlo replica *ℓ* is then defined as

7 $${I}_{\mathrm{CLB}}^{(\ell )}\left(E,\mathrm{ln}{N}_{\mathrm{tot}}\right)=\left\{\begin{array}{ll}\exp \left[{\xi }^{(\ell )}\left(E,\mathrm{ln}{N}_{\mathrm{tot}}\right)\right], & E < {E}_{{\rm{I}}},\\ {A}^{(\ell )}\,{E}^{-{r}_{\mathrm{eff}}^{(\ell )}}, & E\ge {E}_{{\rm{I}}},\end{array}\right.$$

where the normalisation constant *A*^(*ℓ*)^ is determined by enforcing continuity of the background prediction at *E* = *E*_I_. This hybrid construction ensures a smooth transition between the data-driven neural-network prediction in the pre-edge region and the physically motivated extrapolation in the signal region.

The ensemble of extrapolated background models obtained from the Monte Carlo replicas yields pixel- and energy-resolved probability distributions for the CLB across the full energy window. These distributions form the basis for uncertainty propagation to background-subtracted spectra and derived physical observables, completing the workflow summarised in Fig. 1c.

### Validation on synthetic data

To validate the proposed CLB inference framework and its uncertainty quantification, we perform systematic closure tests ^23^ on synthetic EELS-SIs for which the ground-truth background is known by construction ^24^. By isolating the background component in the signal-background decomposition, Eq. (4), these tests provide a controlled assessment of three key aspects: (i) reconstruction accuracy in the pre-edge training window, (ii) statistical calibration of the inferred uncertainties, and (iii) the behaviour of uncertainties in the extrapolation region where no training data are available.

Synthetic spectrum images are generated using power-law backgrounds with spatially varying amplitudes and exponents, combined with controlled noise levels and spatial correlations representative of experimental EELS data. Details of the synthetic data generation, including noise modelling, parameter ranges, and the implementation of spatial correlations, are provided in the Methods section. This validation strategy follows standard closure-testing practice in uncertainty-aware ML, allowing agreement between inferred and true backgrounds to be interpreted statistically.

**Fig. 2:**
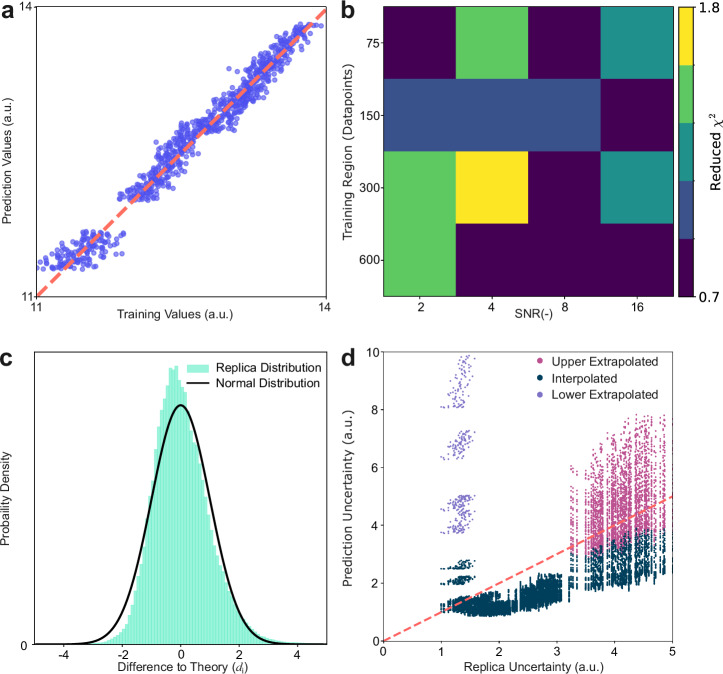
Methodology validation with synthetic data. **a** Scatter plot of the NN model predictions for the CLB, Eq. (7), in the training region as a function of the corresponding synthetic data. The good agreement with the dashed red line (*ρ* = 1) indicates the faithfulness of the training. **b** Qualitative overview of the goodness of fit (reduced *χ*^2^) for a representative set of synthetic EELS-SIs with different assumed SNR values and numbers of training points. **c** Histogram of the differences between NN predictions and training data of (**a**) in units of the corresponding cluster variance, Eq. (8). The agreement with the reference univariate Gaussian demonstrates that the NN predictions fluctuate around the central values by an amount consistent with the underlying EELS-SI noise. **d** Same as (**a**) now for the corresponding NN model (*σ*_NN_) and MC replica (*σ*_MC_) uncertainties. Different colours indicate whether the prediction is interpolated in the total integrated intensity *N*_tot_ (i.e. within the range of cluster-centre values used in training) or extrapolated below or above that range (`lower extrapolated' and `upper extrapolated', respectively). For the bulk of the data, we find *σ*_NN_ ≪ *σ*_MC_, indicating that the neural network model is learning the underlying law. In the extrapolation regions, instead, one finds *σ*_NN_ ≫ *σ*_*MC*_, indicating that the Monte Carlo ensemble of NNs is aware of the limited constraints and hence the ensemble appropriately reflects reduced constraints in the extrapolation region.

Figure 2 summarizes the results of closure tests performed on synthetic EELS-SIs. These tests assess reconstruction accuracy in the pre-edge training region, statistical calibration of the inferred uncertainties, and the behaviour of uncertainty estimates when extrapolating into the signal region beyond the training window, each of which is discussed in turn.

#### Reconstruction accuracy

We first assess the accuracy with which the proposed framework reconstructs the CLB in the pre-edge training region, where the ground-truth background is known by construction. Figure 2a displays a scatter plot of the neural-network predictions for the CLB, Eq. (7), as a function of the corresponding synthetic training data. The strong correlation and the absence of systematic trends indicate that the learned background reproduces the underlying behaviour within the pre-edge region, with residual fluctuations set by the imposed noise level.

Reconstruction accuracy is quantified using the reduced *χ*^2^ between the predicted and true CLB across the pre-edge window. Fig. 2b provides a qualitative overview of the reduced *χ*^2^ obtained for a representative set of synthetic EELS-SIs with different SNRs and numbers of training points. Overall, the reduced *χ*^2^ remains of order unity across much of the sampled range, indicating statistically consistent reconstruction within the assumed noise model, while some configurations show larger deviations as reconstruction becomes more challenging. Because only a coarse sampling of parameter space is shown, Fig. 2b is intended as an illustrative summary rather than a precise determination of the SNR threshold.

#### Statistical calibration and extrapolation uncertainty

Beyond reconstruction accuracy, a central requirement of uncertainty-aware background subtraction is that the predicted uncertainties are statistically calibrated. This calibration is assessed by analysing the distribution of normalised residuals between the predicted and true CLB values in the pre-edge training region, as well as by examining the behaviour of the inferred uncertainties in regions not constrained by training data.

To assess the statistical interpretation of the predicted model uncertainties, Fig. 2c shows the histogram of the normalised residuals *d*_*i*_ between the neural-network predictions and the synthetic training data of Fig. 2a, expressed in units of the corresponding cluster-wise standard deviation,

8 $${d}_{i}^{(\ell ,k)}=\frac{\mathrm{ln}\left({I}^{(k,\ell )}({E}_{i})\right)-{\xi }^{(\ell )}\left({E}_{i},\mathrm{ln}{N}_{\mathrm{tot}}^{(k)}\right)}{{\sigma }_{I}^{(k)}({E}_{i})},\,i=1,\ldots ,{n}_{E},\,k=1,\ldots ,{n}_{K},\,\ell =1,\ldots ,{N}_{\mathrm{rep}}.$$

The agreement of this distribution with a reference univariate Gaussian indicates that fluctuations of the neural-network predictions around the ground-truth background are consistent with the assumed noise model, demonstrating statistically calibrated uncertainty estimates in the training region. The faithfulness of the Monte Carlo replica generation, including the accurate reproduction of both mean spectra and associated uncertainty bands, is further demonstrated in Fig. S2.

Figure 2d shows the same comparison as Fig. 2a, now expressed in terms of the uncertainties predicted by the neural-network ensemble, *σ*_NN_, as a function of the corresponding Monte Carlo replica uncertainties, *σ*_MC_. Here, “interpolated” points denote predictions for values of the total integrated intensity lying within the range spanned by the cluster-centre values used in training, whereas “lower extrapolated” and “upper extrapolated” denote predictions for values below the minimum or above the maximum cluster-centre value, respectively. In the latter cases, the model must extrapolate in the conditioning variable $ln{N}_{{\rm{tot}}}$, which leads to different uncertainty behaviour from the interpolated regime. For the bulk of the training data, we observe *σ*_NN_ ≪ *σ*_MC_, indicating that the neural-network ensemble successfully captures the underlying background behaviour while reducing statistical fluctuations through learning ^25^. In contrast, in the extrapolation region outside the range of cluster-centre values used in training, *σ*_NN_ ≫ *σ*_MC_, reflecting the reduced experimental constraints and demonstrating that the ensemble appropriately reflects reduced constraints in the extrapolation region ^26^. Additional validation of the Monte Carlo replica generation is provided in Fig. S2.

**Fig. 3:**
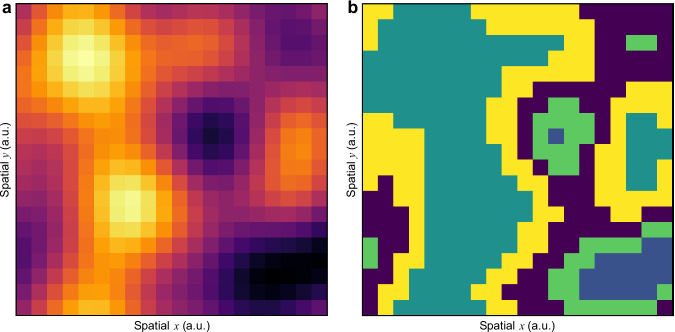
Clustering validation. **a** Seed map used to generate a representative synthetic EELS-SI, with varying colours indicating varying seed values. **b** The resulting *K*-means clustering map, with each colour representing a separate cluster. The high correlation between the two maps demonstrates that the algorithm identifies the intensity variations present in the synthetic EELS-SI.

The performance of *K*-means clustering is validated in Fig. 3, where Fig. 3a displays the seed map used to generate a representative synthetic EELS-SI and Fig. 3b the resulting clustering map. The strong correlation between the original seed map and the resulting cluster one demonstrates that the adopted *K*-means clustering algorithm can accurately recover the variations present in the synthetic EELS-SI. While these closure tests establish the statistical calibration and extrapolation behaviour of the framework under controlled conditions, a critical assessment requires application to experimental data, where noise correlations, background variability, and signal overlap are not known a priori.

To probe the robustness of the closure-test validation beyond the ideal power-law case, we also generate synthetic spectra from a power-law background convoluted with a representative low-loss spectrum. This low-loss spectrum contains a dominant plasmon-like feature together with a weaker higher-energy structure, thereby inducing plural-scattering-like distortions in the resulting CLB. As demonstrated in Fig. S7, the closure test is also successful under this alternative hypothesis for the CLB.

### Application to twisted CrSBr layers

We apply the uncertainty-aware framework to experimental EELS-SIs acquired from twisted CrSBr bilayers, a system that provides a stringent benchmark for resolving weak, spatially periodic core-loss modulations in the presence of background extrapolation uncertainty. CrSBr is particularly well suited for this purpose because small-angle twisting between layers gives rise to a well-defined moiré superlattice, while the associated variations in core-loss spectral features are subtle and therefore highly sensitive to background-subtraction artefacts. The presence of an independently observable structural modulation enables a direct comparison between real-space contrast in STEM and statistically inferred variations in core-loss EELS spectra.

**Fig. 4:**
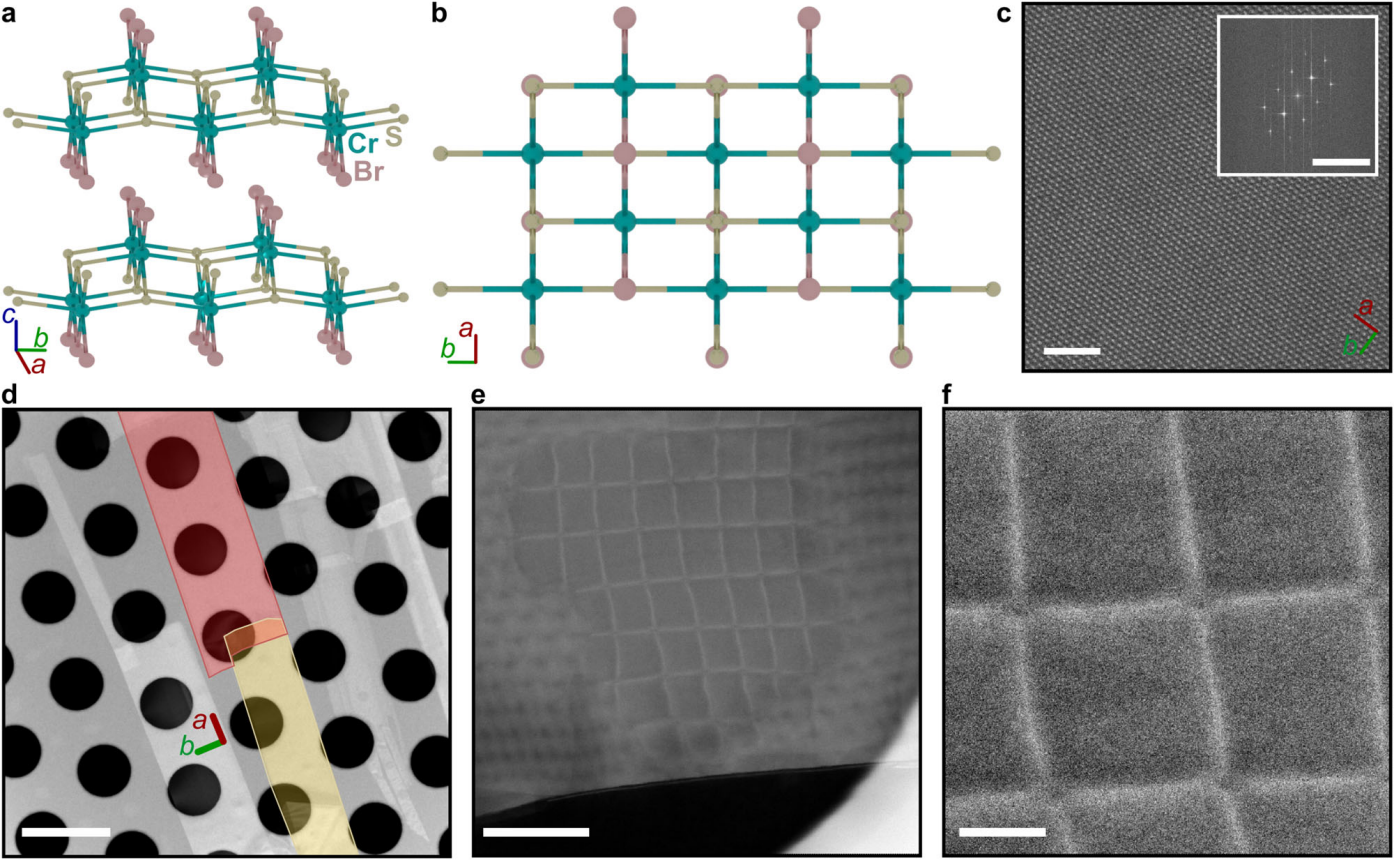
Overview of the CrSBr bilayer specimen. **a** Atomic model of two overlapping layers of CrSBr. **b** Same atomic model as in (**a**), now imaged perpendicular to the layer surface. **c** HR-STEM HAADF image of a CrSBr flake. The inset displays the corresponding FFT, confirming the structural composition of the specimen. **d** Two CrSBr layers (highlighted in false colour) placed on top of a holey SiN grid suitable for TEM inspection. These two CrSBr layers overlap in the central region, resulting in a twisted bilayer configuration. **e** HAADF image of the overlap region of the two coloured flakes of (**d**), displaying a characteristic rectangular moiré periodicity. **f** Magnification of (**e**) focusing on a region with a 3 × 3 moiré cell. The scale bars correspond to **c** 10 nm, inset **c** 10 nm^−1^, **d** 2 *μ*m, **e** 100 nm, and **f** 20 nm.

Figures 4a, b display the atomic structure of two untwisted CrSBr layers in side and top view, highlighting the orthorhombic symmetry of the lattice. A high-resolution HAADF-STEM image of the individual CrSBr flake is shown in Fig. 4c, with the corresponding indexed Fast Fourier Transform (FFT) confirming the crystalline quality and phase purity of the material. Two exfoliated CrSBr flakes were subsequently stacked with a small relative twist and transferred onto a holey SiN TEM grid (Fig. 4d), forming a twisted bilayer in the overlap region with a twist angle of ${\left(0.6\pm 0.1\right)}^{\circ }$. An atomic-scale simulation confirming the correspondence between a ~40 nm moiré periodicity and a twist angle of ≈0. 5°, as shown in Fig. S3.

The HAADF-STEM image of the overlap region (Fig. 4e) reveals a rectangular moiré superlattice, consistent with the reduced rotational symmetry of orthorhombic CrSBr. A magnified view of a representative 3 × 3 moiré cell is shown in (Fig. 4f). This spatially periodic structural pattern provides a natural reference for evaluating whether corresponding variations can be detected in core-loss EELS with quantified statistical confidence.

**Fig. 5:**
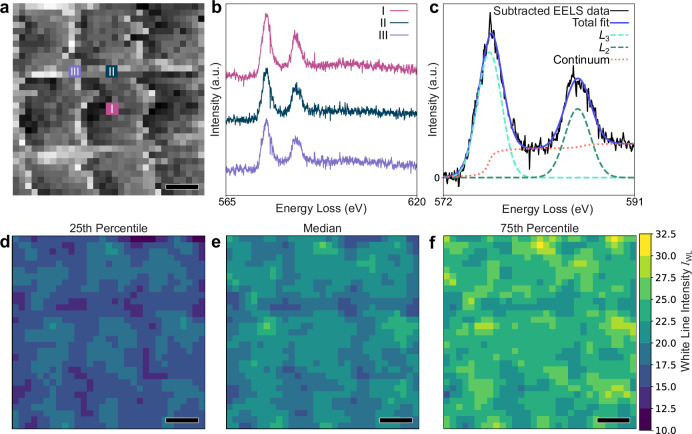
Correlation between moiré periodicity and spatial modulation of core-loss white lines. **a** HAADF image associated with the EELS-SI taken on the twisted CrSBr layers of Fig. 4e, displaying the characteristic moiré periodicity. Three distinct regions of the moiré superlattice are indicated with (I, II, III). **b** Individual spectra associated with the regions labelled with I, II, and III in (**a**), each of them obtained by adding up the contribution from the four indicated pixels in each region. The three spectra are vertically offset to facilitate visualisation. **c** Representative example of the results of fitting Eq. (9) to subtracted core-loss EELS spectra. The separate contributions from the *L*_2_ and *L*_3_ Cr edges and from the continuum states are shown. **d**, **e**, **f** The 25th percentile, median and 75th percentile, respectively, for the total integrated white line intensity, Eq. (10), evaluated across the EELS-SI of (**a**). The spatial modulation of *I*_WL_ follows that of the moiré periodicity observed in the corresponding HAADF image, an effect which remains robust upon accounting for the full inferred uncertainty range. The scale bar in (**a**, **d**, **e**, **f**) corresponds to 40 nm.

Core-loss EELS-SIs were acquired simultaneously with the HAADF-STEM data over the same field of view (Fig. 5a), capturing the Cr L_2,3_ edges in the 570–590 eV range across multiple moiré periods. From this dataset, representative spectra were extracted from three distinct regions of the moiré unit cell, labelled I, II, and III in Fig. 5a. The corresponding averaged spectra (Fig. 5b) exhibit pronounced Cr L_2,3_ white lines, with systematic variations in relative intensity between the selected regions that are comparable in magnitude to the experimental uncertainty.

The full uncertainty-aware background subtraction workflow described in Fig. 1 was applied to the complete EELS-SI. An ensemble of *N*_rep_ = 1000 neural networks was trained on the Monte Carlo replicas generated from the clustered pre-edge background. The resulting model was then evaluated for each spectrum and extrapolated into the signal region using the effective–exponent constraint. The resulting background-subtracted spectra were fitted using a physically motivated model given by

9 $${I}_{{\rm{fit}}}(E)=\mathop{\sum }\limits_{i=2}^{3}\left[{A}_{i}\,{e}^{-{(E-{\mu }_{i})}^{2}/{\sigma }_{i}^{2}}+{C}_{i}\left(\arctan \frac{E-{x}_{0,i}}{{w}_{i}}+\frac{\pi }{2}\right)\right]\,,$$

and consisting of two Gaussian white-line contributions (first term) and continuum contributions (second term). From the fit results, the total integrated white-line intensity normalised to the continuum amplitude can be extracted,

10 $${I}_{{\rm{WL}}}=\frac{{A}_{2}{\sigma }_{2}+{A}_{3}{\sigma }_{3}}{{C}_{2}+{C}_{3}},$$

whose uncertainties are obtained by propagating the full Monte Carlo ensemble through the non-linear fitting procedure.

A representative example of the fit to a background-subtracted spectrum is shown in Fig. 5c, demonstrating excellent agreement between the model and experimental data. Figure 5d–f show spatial maps of the integrated white-line intensity corresponding to the 25th percentile, median, and 75th percentile of the inferred probability distribution. The median map reveals a clear spatial modulation of the Cr L_2,3_ white-line intensity that closely follows the moiré superlattice observed in the HAADF-STEM image.

Importantly, this modulation remains robust across the full uncertainty range. The spatial contrast persists well beyond the propagated confidence intervals, as evidenced by the separation between the lower and upper percentile maps. This demonstrates that the observed variations cannot be attributed to background-subtraction bias, fitting instability, or noise amplification, but instead correspond to statistically significant modulations in the core-loss signal.

While a detailed microscopic interpretation of the Cr L_2,3_ white-lines modulation lies beyond the scope of this work, the observed correlation between moiré periodicity and core-loss intensity is consistent with prior reports of stacking-dependent electronic effects in twisted CrSBr bilayers. Variations in interlayer registry are known to modify local electronic environments, which are sensitively probed by core-level spectroscopy. Here, the emphasis is on the methodological outcome: the ability to detect and quantify such spatially modulated core-loss contrast with rigorous uncertainty control. Overall, this application demonstrates that the proposed framework enables quantitative, nanoscale-resolved core-loss EELS analysis in complex van der Waals heterostructures, even when the relevant spectral variations are weak and inseparable from background extrapolation without statistically controlled inference.

A comparison with a conventional parametric background subtraction approach is provided in Figs. S4 and S5a–c. In particular, this additional analysis shows that a direct pixel-by-pixel parametric subtraction does not resolve the moiré modulation in the white-line intensity map (Fig. S4), whereas a cluster-wise parametric variant combined with the same uncertainty-aware Monte Carlo framework recovers the same qualitative modulation, as shown in Fig. S5a–c, albeit with reduced contrast.

## Discussion

In this work, we have developed a Monte Carlo-based ML framework that enables uncertainty-aware background subtraction in core-loss EELS. By reformulating background modelling as a statistically controlled inference problem, and by combining replica-based learning with physics-informed extrapolation constraints, the approach provides pixel- and energy-resolved background probability distributions rather than a single deterministic estimate. Validation on synthetic datasets demonstrates faithful background reconstruction, statistically calibrated uncertainties, and correct behaviour in extrapolation regions where no training data are available.

Applied to experimental core-loss EELS-SIs acquired from twisted CrSBr bilayers, the framework enables quantitative, nanoscale-resolved mapping of Cr L_2,3_ white-line intensities with rigorous uncertainty propagation. The resulting spatial maps reveal modulations of the core-loss signal that follow the underlying moiré superlattice observed in HAADF-STEM. Importantly, these modulations are robust with respect to the inclusion of the full inferred uncertainty range, demonstrating that they cannot be attributed to background-subtraction bias, fitting instability, or noise amplification.

This application establishes the proposed method as a robust benchmark tool for assessing subtle, spatially modulated core-loss contrast in complex vdW heterostructures, where background extrapolation and uncertainty quantification are critical. A current limitation of the approach is that it requires a sufficiently clean separation between the pre-edge background region used for training and the signal region. If signal and background cannot be reliably separated, physical signal contributions may enter the training data and bias the inferred background. This limitation is not specific to the present method, but is shared more generally by background-subtraction approaches based on a pre-edge fit window. More broadly, the framework provides an automation-ready pathway for quantitative, uncertainty-aware analysis of core-loss EELS data, with direct relevance to a wide range of spectroscopic imaging problems in low-dimensional and quantum materials. Here, automation-ready means that the same workflow, including preprocessing, cluster-wise replica generation, training, extrapolation, and uncertainty propagation, can be applied to different specimens with minimal human intervention and without case-by-case redesign of the background-subtraction methodology.

Beyond the specific application to CrSBr white lines studied in this work, the methodology is readily extendable to other core-loss edges, low-loss regimes, and related spectroscopic techniques, including X-ray absorption and cathodoluminescence spectroscopy. Its generality is further illustrated by an additional application to an independent hBN dataset, presented in Fig. S6. In particular, uncertainty-aware core-loss EELS could be particularly useful for heterointerfaces, where the relevant physics often appears as small changes in ELNES, edge onset, or white-line ratios over only a few atomic planes, and for beam-sensitive specimens, where dose-limited acquisition produces low-SNR spectra that make background subtraction and quantitative interpretation especially delicate. As such, our method offers a general strategy for incorporating statistically rigorous uncertainty quantification into spectroscopic analysis workflows, enabling more reliable interpretation of weak spectral signatures in increasingly complex materials systems.

## Methods

### Data preprocessing and region definition

An EELS spectral image (EELS-SI) can be represented as an *n*_x_ × *n*_y_ grid, where each pixel (*i*, *j*) corresponds to an individual EELS intensity spectrum *I*^(*i*, *j*)^(*E*) binned in the energy-loss value *E*. Before the CLB subtraction method can be applied to these EELS-SI, a pre-processing treatment is required. This data preprocessing contains three steps: spectral alignment, data pooling, and region definition.

### Spectral alignment

Due to spectral drift, EELS-SI may fall out of alignment. There are two main ways to align them, either using the zero-loss peak (ZLP) as a calibration reference or using cross-correlation with a reference spectrum.

Spectral alignment using the ZLP is performed by shifting each individual spectrum composing the EELS-SI by an amount ${E}_{{\rm{shift}}}^{(i,j)}$ defined as:

11 $${E}_{{\rm{shift}}}^{(i,j)}={\rm{argmax}}({I}_{{\rm{low-loss}}}^{(i,j)}(E)),$$

with ${I}_{{\rm{low-loss}}}^{(i,j)}(E)$ indicating the EELS intensity in the low-loss region. In this procedure, one shifts individual spectra to ensure that the maximum of the ZLP is located at *E* = 0. Alignment by ZLP is optimal, but it is not always feasible due to requiring the simultaneous acquisition of spectra in the low-loss and core-loss regions. Alternatively, spectral alignment by cross-correlation with a reference spectrum does not require low-loss data acquisition, but it can result in misalignment due to real physical shifts. In this method, spectral shifts are obtained by choosing a reference spectrum with index (*i*_ref_, *j*_ref_), then spectrally shifting the remaining spectra by:

12 $${E}_{{\rm{shift}}}^{(i,j)}={\rm{argmax}}({I}^{({i}_{{\rm{ref}}},{j}_{{\rm{ref}}})}(E)\times {I}^{(i,j)}(E))\,,$$

such that the shift corresponds to the energy where the cross correlation with the reference spectrum is at a maximum.

### Data pooling

The signal-to-noise (SNR) of the acquired data can be improved by pooling the SI with a pre-defined kernel *m*. The pooled data is then the convolution of this kernel with each energy bin of the SI. Pooling combines information from neighbouring pixels, whose spatial correlation is used to reduce statistical fluctuations.

### Training region definition

Before the ML model for the CLB can be trained, one needs to specify which regions in energy loss *E* are assigned to the training dataset and which ones instead are classified as signal regions where the model must be extrapolated. In the latter, the model is extrapolated using the effective exponent method discussed in the main manuscript. These two regions are defined by three parameters: $[{E}_{\min },{E}_{{\rm{I}}}]$ for the training region, and $[{E}_{{\rm{I}}},{E}_{\max }]$ for the extrapolation (signal) region, as schematically shown in Fig. S1. Only energy-loss values in $[{E}_{\min },{E}_{{\rm{I}}}]$ are used to train the model, where *E*_*I*_ is chosen to avoid any relevant signal features, such as the Cr edges discussed in the main manuscript. The effective exponent region is defined by a window of Δ*E* = 5 eV just before the onset of the extrapolation region. Our choice of Δ*E* = 5 eV for the energy window region is suitable for estimating the logarithmic slope that controls the extrapolation in this specific specimen, rather than as a universal standard value. In general, the chosen value of Δ*E* should be wide enough to reduce sensitivity to channel-to-channel noise, but narrow enough to remain representative of the background behaviour immediately below the edge onset, and Δ*E* = 5 eV satisfies both conditions.

### Physical motivation for the extrapolation form

The usage of the term “physics-informed” in the present work does not imply that physical constraints are built directly into the neural-network architecture itself. Rather, the physics input enters at the extrapolation stage, when making model predictions in the signal region. In core-loss EELS, the pre-edge background is commonly described, over a limited energy range, by a smooth monotonically decaying function, often well approximated by a power law. For this reason, once the background has been learned in the signal-free pre-edge region, we do not allow the neural network to extrapolate arbitrarily into the ionisation-edge window. Instead, the continuation above *E*_*I*_ is constrained to follow a power-law form. The exponent of this power-law continuation is not fixed a priori, but is estimated from the local logarithmic slope of the learned background immediately below the edge onset. In this way, the extrapolation remains adaptive to local spectral variations present in the data, while still being restricted to a functional form that reflects the expected behaviour of the CLB. The resulting procedure is therefore hybrid: the value of the effective exponent is data-driven, whereas the admissible form of the extrapolation is physically motivated. This is the sense in which we use the term “physics-informed” in the manuscript. The constraint is not introduced as a post-hoc sanity check, but as a regularisation of an otherwise ill-posed extrapolation problem in a region where no training data are available. Matching the power-law continuation to the neural-network prediction at *E*_*I*_ enforces continuity of the inferred background, while the use of the local logarithmic slope ensures that the extrapolated decay remains consistent with the behaviour learned immediately below the edge onset.

### Synthetic data generation for closure tests

Synthetic EELS-SIs are generated to validate the proposed CLB inference framework under controlled conditions, where the ground-truth background is known by construction. For the purposes of methodology validation, only the background component of the core-loss EELS-SI is simulated, allowing reconstruction accuracy and uncertainty calibration to be assessed independently of inelastic signal contributions.

Each synthetic EELS-SI consists of an (*n*_*x*_, *n*_*y*_, *n*_*E*_) spatial grid, where each pixel is associated with an individual spectrum sampled over *n*_*E*_ energy-loss channels. In the validation presented in the main text, we use *n*_*x*_ = *n*_*y*_ = 20, yielding a total of 400 spectra, each spanning the energy-loss from *E*_0_ = 300 eV to *E*_*f*_ = 600 eV with *n*_*E*_ = 900 energy bins.

The CLB intensity at each pixel is modelled using a power-law form,

13 $$I(E)=A{\left(E/{E}_{0}\right)}^{-r},$$

where *A* denotes the background amplitude at the onset energy *E*_0_, and *r* is the power-law exponent. To capture realistic spatial variability, the parameters *A* and *r* are sampled independently for each pixel, with *A* ∈ [10^5^, 10^6^] and *r* ∈ [2, 3], ranges chosen to reflect typical experimental core-loss EELS spectra.

Spectral noise in experimental core-loss EELS arises from multiple sources whose individual contributions are difficult to characterise. By the central limit theorem, these combined fluctuations can be approximated by a Gaussian distribution. Accordingly, Gaussian noise is added to each synthetic spectrum, with the variance chosen to reproduce a prescribed per-pixel SNR in the pre-edge energy window. Specifically, the noise contribution is drawn from

14 $${I}_{{\rm{noise}}}(E) \sim {\mathcal{N}}\left\{0,\frac{\frac{1}{{n}_{E}}\sqrt{{\sum }_{E={E}_{0}}^{{E}_{f}}{\left(I(E)\right)}^{2}}}{{\rm{SNR}}}\right\}.$$

and added to the underlying power-law background.

To incorporate spatial correlations in the noise and background parameters, a seed map with the same spatial dimensions as the EELS-SI is generated using Perlin noise, a form of gradient noise that is inherently spatially correlated. The seed map is then used to generate correlated variations of the power-law parameters according to

15 $$\begin{array}{rcl}A & = & {A}_{\min }+{P}_{{\rm{seed}}}({A}_{\max }-{A}_{\min }),\\ r & = & {r}_{\min }+{P}_{{\rm{seed}}}({r}_{\max }-{r}_{\min }).\end{array}$$

where *P*_seed_ denotes the Perlin-noise value at each pixel. This procedure ensures that both the background amplitude and decay exponent vary smoothly across the synthetic EELS-SI, mimicking experimentally observed thickness- and density-related variations. The resulting synthetic datasets therefore capture the key statistical features of experimental core-loss EELS-SIs, including realistic background shapes, controlled noise levels, and spatial correlations, providing a robust testbed for validating background reconstruction accuracy and uncertainty propagation.

### ML model uncertainty estimate

Once the ML training has been completed, we end up with an ensemble of neural network predictions *I*^(*n*)^(*E*), with *n* = 1, …, *N*_rep_, characterising the CLB from which statistical estimators such as means, variances, and correlations can be readily evaluated. Specifically, for every energy channel *E*, the mean prediction $\bar{I}(E)$ and the associated standard deviation *σ*_*I*_(*E*) are computed as

16 $$\bar{I}(E)=\frac{1}{{N}_{\mathrm{rep}}}\mathop{\sum }\limits_{n=1}^{{N}_{\mathrm{rep}}}{I}^{(n)}(E),\,{\sigma }_{I}(E)=\sqrt{\frac{1}{{N}_{\mathrm{rep}}-1}{\sum }_{n=1}^{{N}_{\mathrm{rep}}}{\left({I}^{(n)}(E)-\bar{I}(E)\right)}^{2}}.$$

Similar relations apply to any other quantity derived from the background-subtracted intensity, such as the white-line intensities *I*_WL_. Specifically, the fit to the background-subtracted intensity using Eq. (9) in the main manuscript and the associated calculation of *I*_WL_ are carried out replica by replica, producing an ensemble ${I}_{{\rm{WL}}}^{(n)}$ with *n* = 1, …, *N*_rep_, from which to evaluate its mean and associated uncertainties in the same manner as for Eq. (16).

### Computational performance

The main bottleneck of the CLB subtraction methodology presented in this work is the training of neural networks on the EELS-SI data replicas, with all other computational steps being negligible in relative terms. This training time is primarily influenced by two factors: the number of clusters in the EELS-SI and the density of data points in the pre-edge region. The computational cost scales approximately linearly with both, meaning that larger cluster counts or denser pre-edge sampling significantly increase the required training time. Nevertheless, for typical EELS-SI datasets such as the ones considered in this work, the training times remain at around 1 min per replica, which, given that replica training can be fully parallelised, indicates that our full workflow can be carried out in a few minutes when run in a reasonable computing cluster. Should this be required in the future, additional speed-ups can be achieved by adapting the workflow to run on GPUs, as demonstrated by a closely related ML application in ref. ^27^.

### Choice of ML hyperparameters

Table 1 collects the values of the hyperparameters used for the ML analysis presented in the main manuscript. The neural network architecture is a multi-layer perceptron with two input neurons and one hidden layer with 16 neurons. For each of the hyperparameters, we indicate their category, the label used to define them in the code, and the numerical value used for the analysis.

**Table 1:** Hyperparameters used for the analysis presented in the main manuscript

Category	Hyperparameter	Value
File	lowloss	False
NN architecture	Type	MLP
	Nodes	2 → 16 → 1
	Activation	SiLU
Energy window	energy_window	(520, 570, 670)
Preprocessing	log_energy	True
	standardize_targets	False
Training	epochs	200
	lr	1 × 10^−3^
	batch_size	100
	patience	100
	min_delta	1 × 10^−4^
	lambda_deriv	10.0
Evaluation	effective_exponent_window_size	5
Pooling	pool_radius	2
	gaussian_kernel	True
Clustering	n_clusters	4

The NN architecture is a multi-layer perceptron with 2 input neurons and one hidden layer with 16 neurons.

### Twisted CrSBr heterostructure fabrication

CrSBr layers were obtained by exfoliating bulk crystals, transferred onto silicon substrates, and then deterministically placed onto TEM grids (PELCO® Holey Silicon Nitride Support Film, 200 nm, 1000 nm pores) using a polycarbonate-assisted method, using the method reported in ref. ^28^. The sample thickness is (30 ± 10) nm, with a twist angle of ${\left(0.6\pm 0.1\right)}^{\circ }$.

### TEM and EELS analyses

Twisted CrSBr bilayers were characterised at 60 keV using an ARM200F Mono-JEOL microscope. The EELS signal was recorded using the CMOS camera of the GIF Continuum HR spectrometer. The convergence and collection semi-angles were 24.6 mrad and 58.4 mrad, respectively, and the aperture of the spectrometer was set to 5 mm. EELS-SIs were acquired with 0.05 eV/channel dispersion and 1000 ms dwell time. The full width at half maximum (FWHM) of the zero-loss peak was 0.6 eV, indicating the achieved energy resolution. In the effective-exponent extrapolation, we use an averaging window of Δ*E*_*r*_ = 5 eV and an energy-bin size of Δ*E*_bin_ = 0.05 eV/channel.

## Supplementary information

Supplementary Information

## Data Availability

The datasets generated and analysed during the current study are available in the GitHub repository, https://github.com/BWielen/EELSfitter-Core_loss. This repository contains the dataset required to interpret, replicate, and build upon the findings reported in this study, including raw EELS-SIs, processed spectra, and metadata. The code implementing the uncertainty-aware CLB subtraction framework described in this work is available at https://github.com/BWielen/EELSfitter-Core_loss under an open-source license.

## Code availability

The code implementing the uncertainty-aware CLB subtraction framework described in this work is available at https://github.com/BWielen/EELSfitter-Core_loss under an open-source license.
