# Supplementary material for: Uncertainty-aware machine learning for core-loss background subtraction in EELS
Source: NPJ Comput Mater. 2026 May 26;12(1):278. doi: 10.1038/s41524-026-02145-3 (PMC13485672; doi:10.1038/s41524-026-02145-3)
Supplement: Supplementary file 1 [file 41524_2026_2145_MOESM1_ESM.pdf]

Uncertainty-Aware Machine Learning for Core-Loss Background Subtraction in EELS

Supplementary Information

Bart van der Wielen[1], Jeroen J. M. Sangers[1], Samuel Mañas-Valero[1],
Juan Rojo[2,3], and Sonia Conesa-Boj[1].

[1]Kavli Institute of Nanoscience, Delft University of Technology, Delft, 2628 CJ, The Netherlands

[2]Nikhef Theory Group, Science Park 105, 1098 XG Amsterdam, The Netherlands

[3]Department of Physics and Astronomy, VU Amsterdam, 1081 HV Amsterdam, The Netherlands

Corresponding author: s.conesaboj@tudelft.nl

This Supplementary Information (SI) provides additional supporting figures and notes related to the ML-based core-loss background (CLB) subtraction methodology presented in the main manuscript.

This Supporting Information provides additional supporting figures related to the ML-based core-loss background (CLB) subtraction methodology presented in the main manuscript. It is organised as follows: Fig. S1 illustrates the training and extrapolation regions; Fig. S2 provides additional validation of the Monte Carlo replica generation; Fig. S3 illustrates the twist-angle determination; Figs. S4 and S5 compare the method with parametric background subtraction; Fig. S6 presents an application to hBN; and Fig. S7 reports closure-test validation with an alternative underlying law.

Training and extrapolation regions

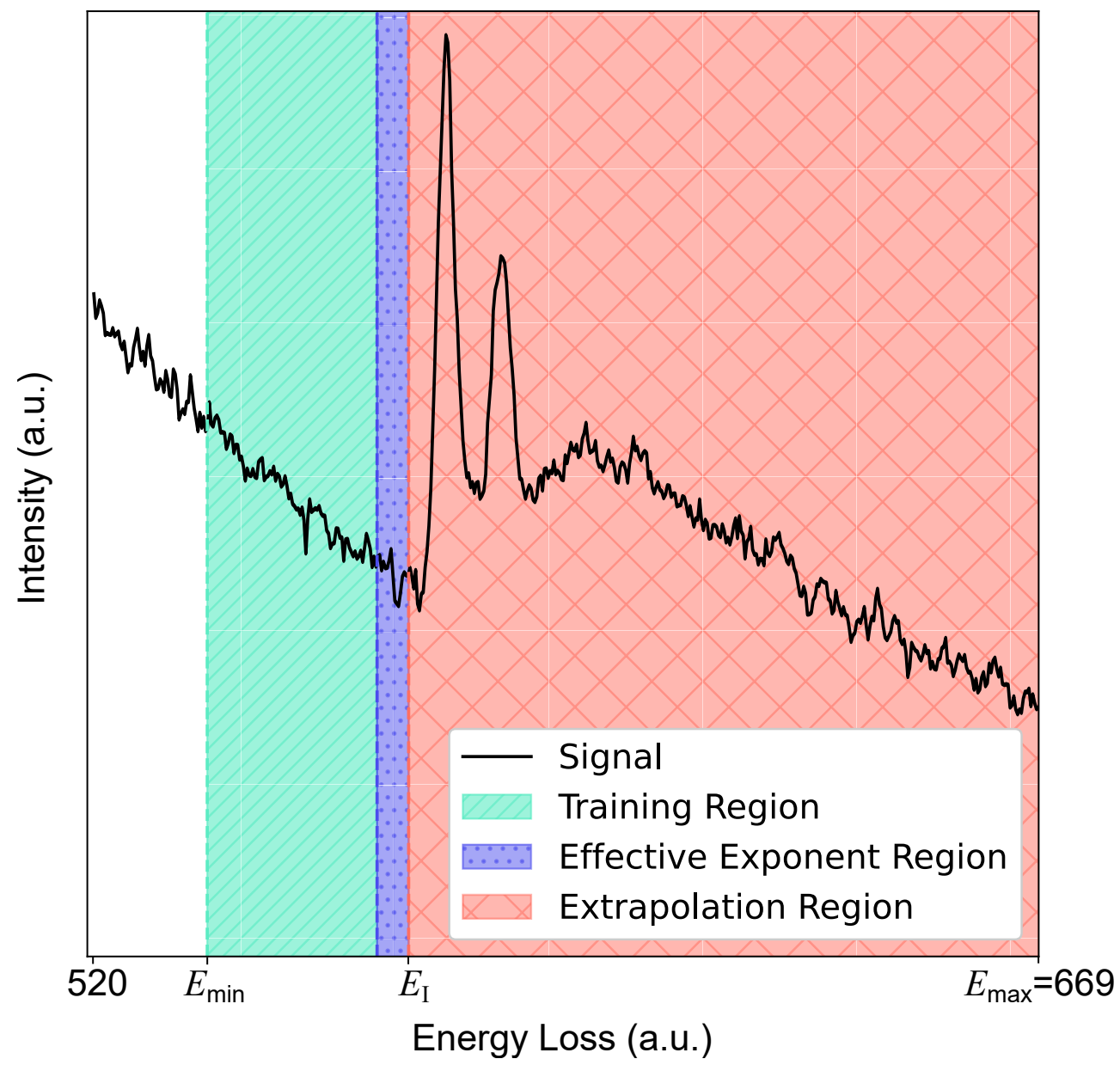

Figure S1: Definition of training and extrapolation regions. The NN model is trained on both the mint-blue line-hatched region and the marine-blue dot-hatched region and corresponds to $[E_{\mathrm{min}}, E_{\mathrm{I}}] = [538, 570]$ eV. The effective exponent determining the extrapolation behaviour of the model in the signal region is computed in the marine-blue dot-hatched region $[565, 570]$ eV. A power-law is extrapolated in the coral cross-hatched extrapolation region defined as $[E_{\mathrm{I}}, E_{\mathrm{max}}] = [570, 669]$ eV.

Validation of MC replica generation

By applying our methodology to the synthetic core-loss EELS-SI generated according to the aforementioned procedure, we can determine its faithfulness and reliability. Here we provide additional details on the validation of the MC replica generation, where we compare representative spectra within a cluster to the $N_{\rm rep} = 1000$ replicas Monte Carlo replicas generated from sampling over the cluster mean and covariance. Fig. S2a compares the mean and the associated uncertainty (evaluated as 90% C.L. intervals) of the core-loss intensity $I(E)$ within this cluster with the corresponding estimators evaluated from the generated Monte Carlo replicas. The excellent agreement both for the mean and for the uncertainties in the full range of energy-loss demonstrate that the adopted sampling faithfully reproduces the original statistical properties of the cluster and therefore can be used as the basis for accurate error propagation of the neural network model.

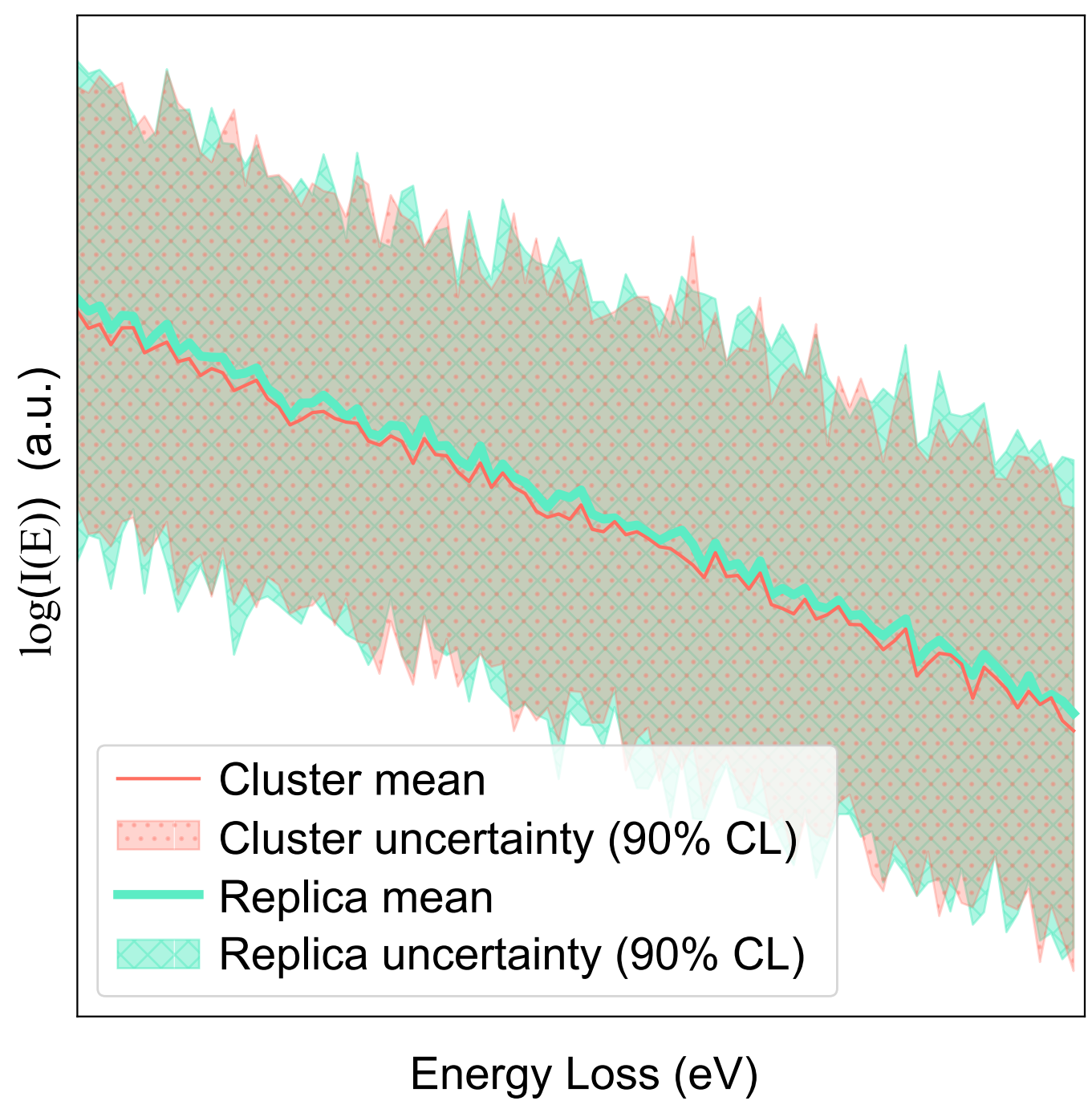

Figure S2: Validation of MC replica generation. Comparison between the mean and the uncertainty (defined as the 90% C.L. interval) of the logarithmic core-loss intensity $I(E)$ within a single cluster in the synthetic EELS-SI (mint cross-hatched) as a function of the energy loss E with the corresponding estimators evaluated from the generated $N_{\rm rep} = 1000$ Monte Carlo replicas (coral dot-hatched). Excellent agreement is obtained in both cases.

Twist angle determination

The twist angle of the sample was geometrically determined using:

$$\sin\theta = \frac{a}{L} \rightarrow \theta \approx \frac{a}{L} \text{ for } \theta \ll 1\,, \tag{1}$$

with θ being the twist angle, L the moiré periodicity, and a the lattice constant. In the case of CrSBr with a rectangular lattice, a can either be 3.54 Å or 4.76 Å [1]. In the twisted CrSBr specimen considered in the main manuscript, the moiré periodicity was found to be $\sim$ 40 nm, which corresponds to a twist angle to be $(0.6 \pm 0.1)^\circ$.

To confirm this geometry-based calculation, Fig. S3 shows a representation of two relatively twisted CrSBr lattices. Fig. S3a displays an atomic resolution simulation of twisted CrSBr with a relative twist angle of 0.5°. Fig. S3b shows the same simulation, now with similar nodes of the moiré periodicity marked. A moiré periodicity of $\sim$ 40 nm is found to correspond to a twist angle of 0.5°, confirming the geometrical calculations based on Eq. (1).

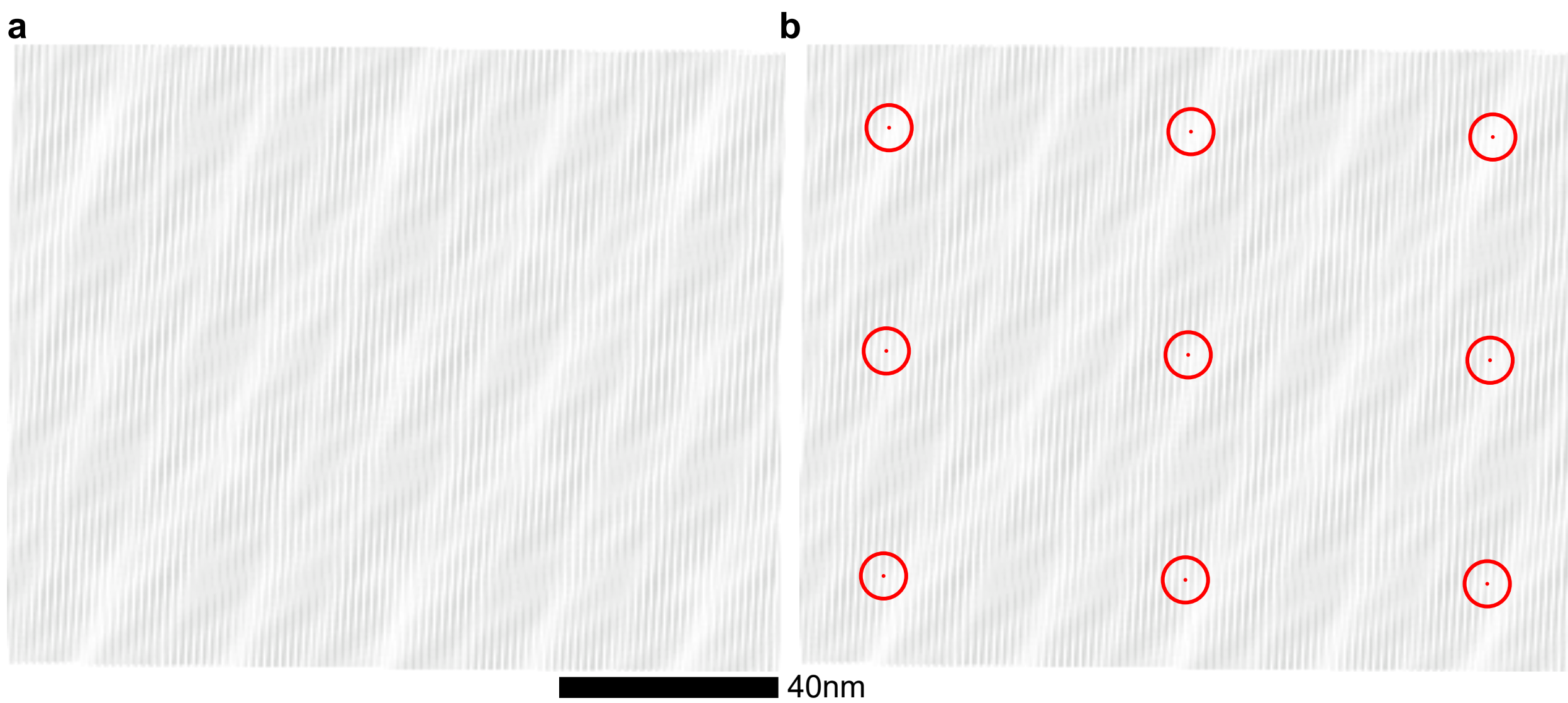

Figure S3: Determination of twist angle in CrSBr. a) Atomic resolution simulation of twisted CrSBr with a relative twist angle of 0.5° **b)** Same as **a)**, now with similar nodes of the moiré periodicity marked. A moiré periodicity of $\sim$ 40 nm is found to correspond to a twist angle of 0.5°, confirming the geometrical calculations based.

Comparison with parametric background subtraction

We can compare our ML approach for EELS core-loss background subtraction to a more traditional parametric approach where this background is modelled using

$$I_{\rm CLB}(E) = AE^{-r}\,, \tag{2}$$

in both the background and signal regions, with A, r parameters to be constrained by the data. Replacing our ML model with such parametric functional form requires that either one drops the smooth interpolation of the thickness dependence of the background (which in our case enters through the proxy $\ln N_{\rm tot}$ in Eq. (7) in the main manuscript), or that one drops the data-driven uncertainty estimate which enters via the clustering procedure. Here we examine the outcome of these two options.

First, we neglect the clustering procedure and hence the associated uncertainty estimate and fit Eq. (9) pixel-by-pixel in the EELS spectral image to the same region as that used for the ML model training. Following the analysis pipeline described in the manuscript, we obtain in Fig. S4 the corresponding white line intensity map, which can be compared to Fig. 5e in the main manuscript. In this case one does not observe the tell-tale Moiré modulation of the white line intensity, indicating that a direct pixel-by-pixel application of the parametric model Eq. (9) is not sensitive enough to resolve the Moiré modulation signal present in our specimen from statistical fluctuations of the data.

As an alternative approach, the parametric model of Eq. (9) can be combined with our data-driven Monte Carlo uncertainty estimate if applied to the cluster-by-cluster replicas. In this case the procedure follows all the steps indicated in Fig. 1 in the main manuscript, and replaces the neural network training by a parametric model fitted to the same region (and that hence can extrapolate to the signal region). The results obtained this way for the white-line intensity map are shown in Fig. S5, which can be compared to Fig. 5d,e,f in the main manuscript. For this specific specimen, one can resolve the characteristic Moiré modulation of this specimen, consistently with the results obtained with our ML approach, although with a somewhat degraded contrast. The main difference between the results of Figs. S4 and S5 is that in the former the lack of uncertainty analysis renders the power-law parametric approach too sensitive to numerical instabilities associated to background noise fluctuations.

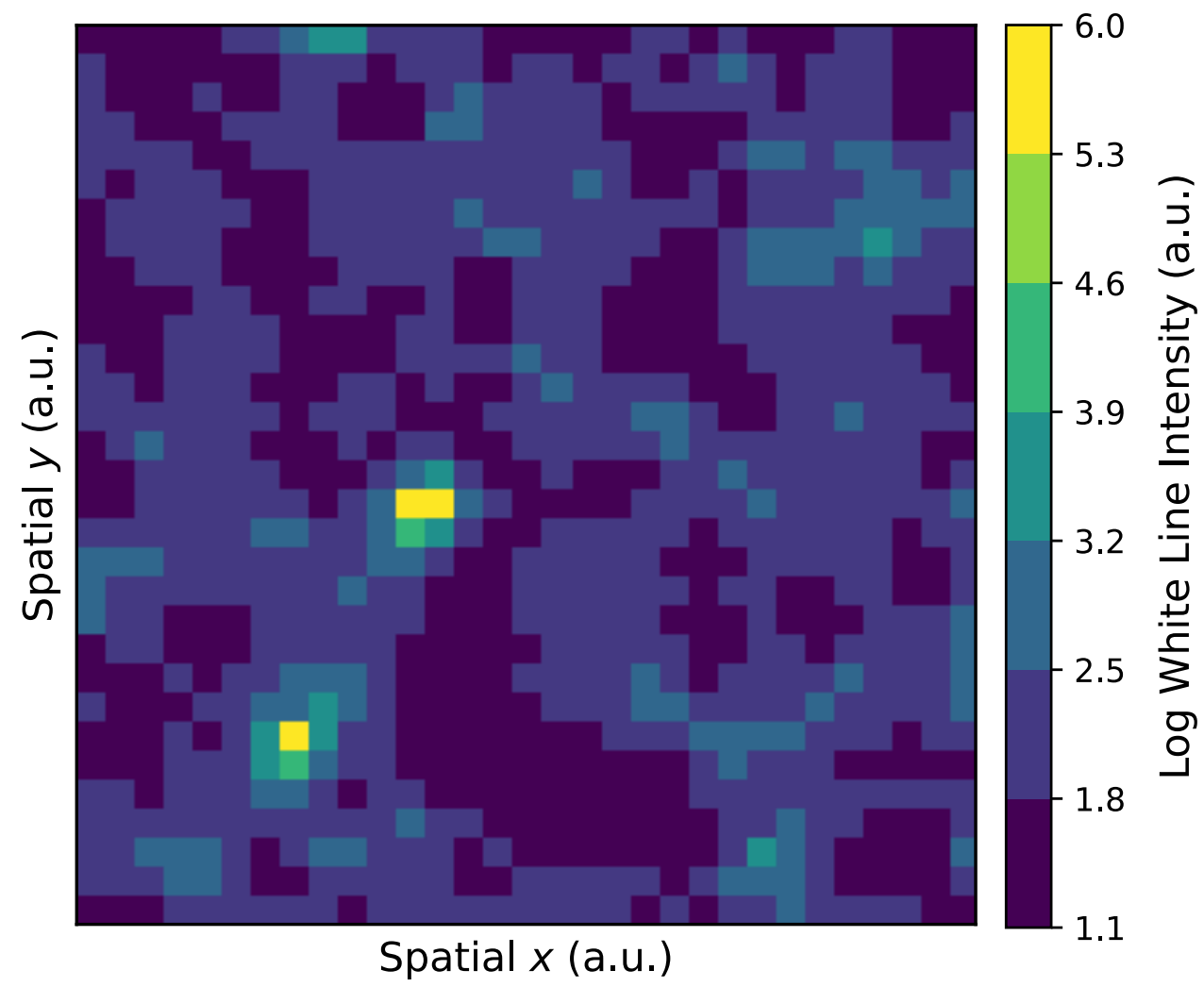

Figure S4: White-line intensity obtained from pixel-by-pixel parametric background model. Same as Fig. 5d in the main manuscript, now with the core-loss background modelled pixel-by-pixel with a parametric model fitted to the same region as that used for the ML model training. Due to the absence of clustering, in this case no uncertainty analysis is possible. Note the logarithmic scale in the colour scale map. No Moiré modulation of the white line intensity is observed when using such a pixel-by-pixel parametric background model.

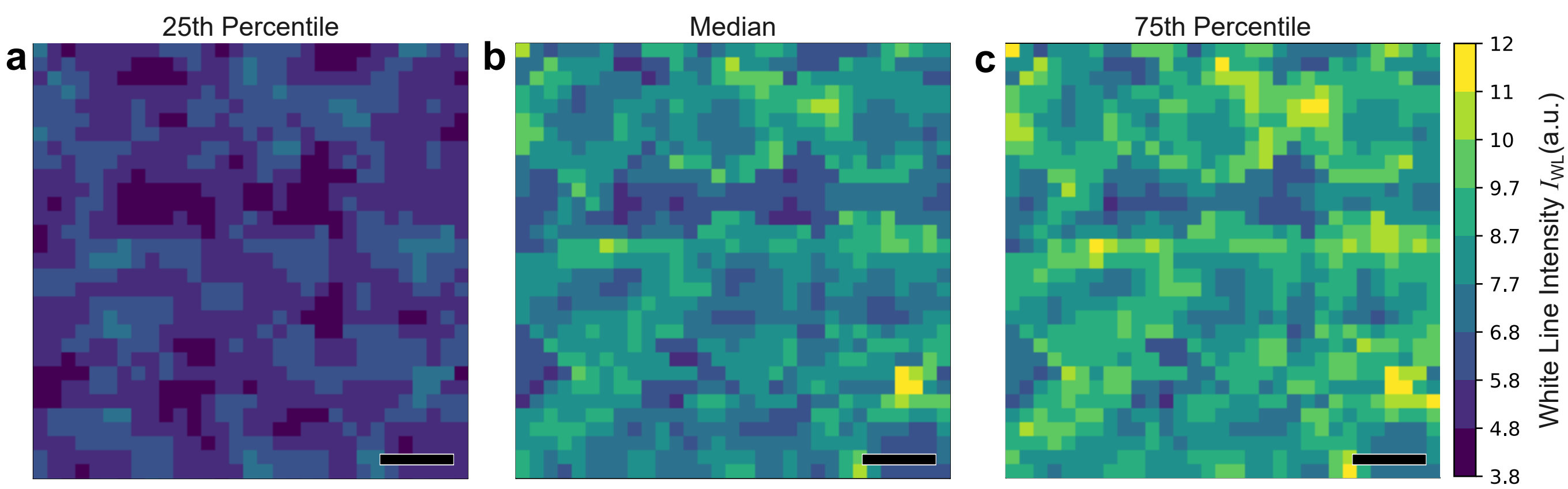

Figure S5: White-line intensity obtained from a cluster-wise parametric background model. a) 25th percentile map of the total integrated white-line intensity I_{WL}. **b)** Median map of I_{WL}. **c)** 75th percentile map of I_{WL}. Same as Fig. 5**d)–f)** in the main manuscript, now with the cluster-wise ML model replaced by a parametric model for the background. In contrast to Fig. S4, this approach retains the data-driven uncertainty estimate in the background model.

Application to a hBN specimen

The flexibility of our EELS core-loss background removal method enables its straightforward application to very different specimens. To illustrate this flexibility, Fig. S6 displays an EELS linescan of 170 nm corresponding to the core-loss region between 180 eV and 230 eV and taken on a monocrystalline hexagonal boron nitride (hBN) specimen located on top of holey silicon nitride. Fig. S6a corresponds to the original measurement while Fig. S6b corresponds to the background subtracted counterpart. The horizontal feature arising in Fig. S6a at a length of around 90 nm corresponds to a bend in the specimen, which is completely removed by background subtraction. Our methodology was successfully applied to this new hBN specimen without having to adjust any of its hyperparameters, therefore demonstrating its robustness.

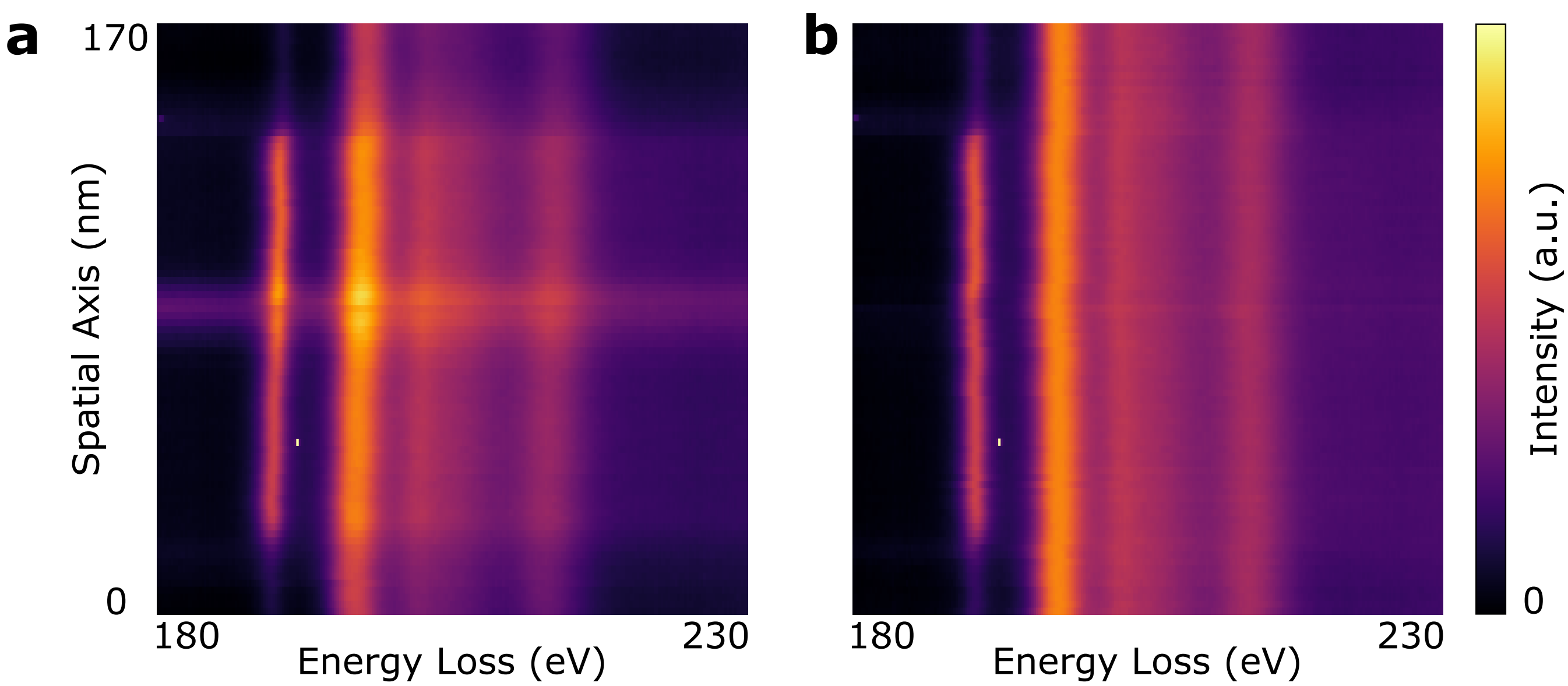

Figure S6: Application of core-loss background subtraction to an EELS linescan of a hBN specimen. a) Original linescan. **b)** Same **a)** with the core-loss background removed using the same methodology described in the main manuscript.

Closure test validation with an alternative underlying law

The closure test validation of our core-loss background removal methodology presented in the main manuscript assumes a power-law behaviour for the background. However, the unique property of feed-forward neural networks as flexible functional approximators implies that statistical closure must also be found should one assume different underlying laws for the core-loss background. To corroborate this expectation, Fig. S7 displays the fit residuals, same as Eq. (8) in the main manuscript, obtained in closure tests carried out with a different assumption for underlying law for the core-loss background. Specifically, we consider now for $I_{\mathrm{CLB}}(E)$ a power-law behaviour convolved with a representative low-loss spectrum containing a dominant plasmon feature together with weaker higher-energy structure, used to mimic plural-scattering-induced distortions of the core-loss background shape.

As in the case of the original closure tests in Fig. 2c, the residuals in Fig. S7 follow an univariate Gaussian distribution, indicating that predictions of the ML model fluctuate around the underlying theory assumed in the closure tests according to the nominal model uncertainties. In other words, the fluctuations of the neural network predictions around the ground-truth background are consistent with the assumed noise model, demonstrating statistically calibrated uncertainty estimates in the training region.

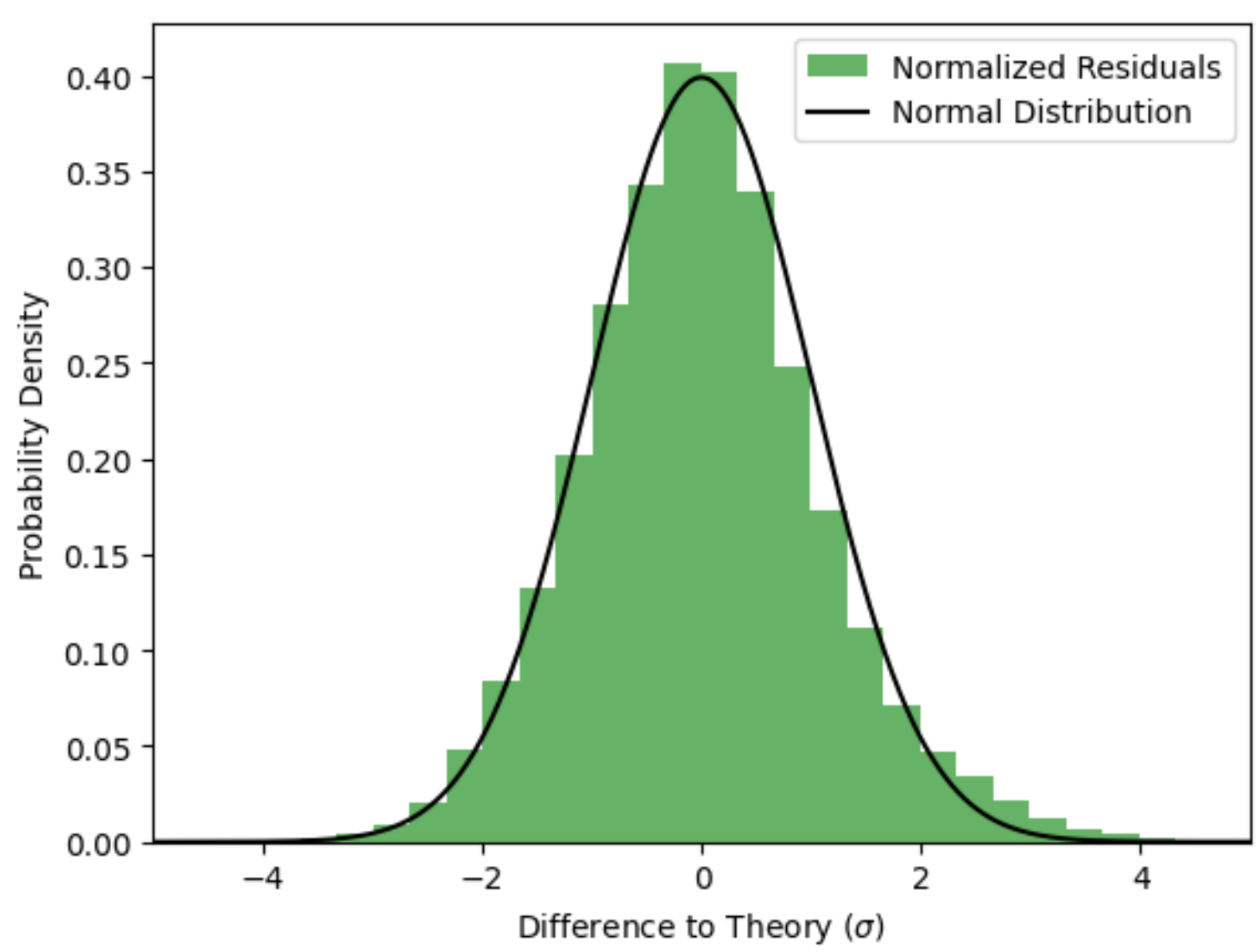

Figure S7: Closure test validation with an alternative underlying law. Same as Fig. 2c in the main manuscript, now obtained in closure tests where synthetic data has been generated with a different underlying law for the core-loss background, namely a power-law behaviour convolved with a low-loss spectrum which contains the contribution from plural scatterings. As in the case of the original closure tests in Fig. 2c, the fit residuals (Eq. (8) in the main manuscript) follow an univariate Gaussian distribution, indicating that predictions of the ML model fluctuate around the underlying theory assumed in the closure tests according to the nominal model uncertainties.

References

[1] Project, M. Materials data on crsbr by materials project (2020). URL https://www.osti.gov/servlets/purl/1199151/.
